# A case of relapsing anti-GBM disease secondary to alemtuzumab therapy

**DOI:** 10.1007/s13730-023-00822-6

**Published:** 2023-11-09

**Authors:** Saskia M. Leibowitz, Valli Manickam, Vikas Srivastava, George Kan

**Affiliations:** 1 Nephrology Department, Townsville Hospital, Douglas, Australia; 2 University of Queensland, Brisbane, Australia

**Keywords:** Alemtuzumab, Anti-GBM disease, Glomerulonephritis

## Abstract

We report the first case of relapsing anti-GBM disease secondary to alemtuzumab in a 24-year-old female with relapsing–remitting multiple sclerosis. Initial anti-GBM disease was detected 10 months after alemtuzumab was given and was diagnosed by demonstrating high anti-GBM antibody titers and with a confirmatory kidney biopsy. The patient presented with a rapidly progressive glomerulonephritis with no pulmonary involvement. After appropriate treatment, the patient went into remission with undetectable anti-GBM antibodies. However, 20 months later, the patient re-presented with relapsing anti-GBM disease. Despite aggressive treatment, the patient became dialysis-dependent.

## Case report

A then-22-year-old female presented to hospital with a week of hematuria and uremic symptoms. The patient had a history of relapsing–remitting multiple sclerosis (MS) that had been treated with fingolimod and one cycle of alemtuzumab 10 months prior. She had a history of migraines and chronic inflammatory cystitis, was a non-smoker and did not drink alcohol.

**Fig. 1:**
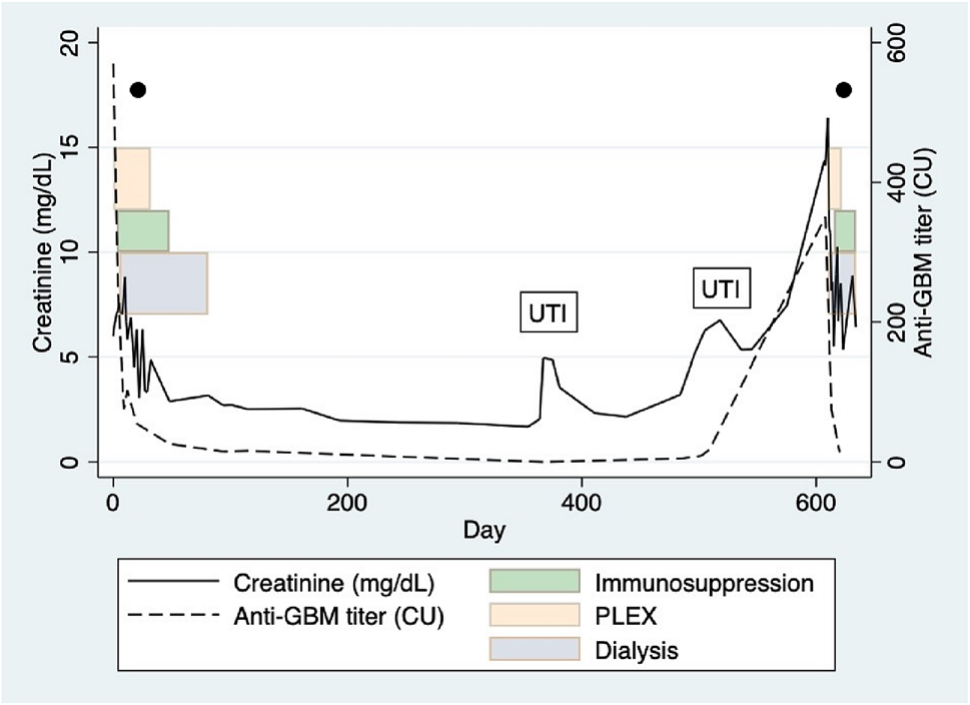
Trend of serum creatinine (mg/dL) and anti-GBM antibody titer (CU) from the first presentation onwards. Periods of apheresis (PLEX), dialysis and induction immunosuppression are highlighted with colored bars. Urinary tract infection (UTI) episodes indicated in text boxes. Timing of the two kidney biopsies indicated by black dots

**Fig. 2:**
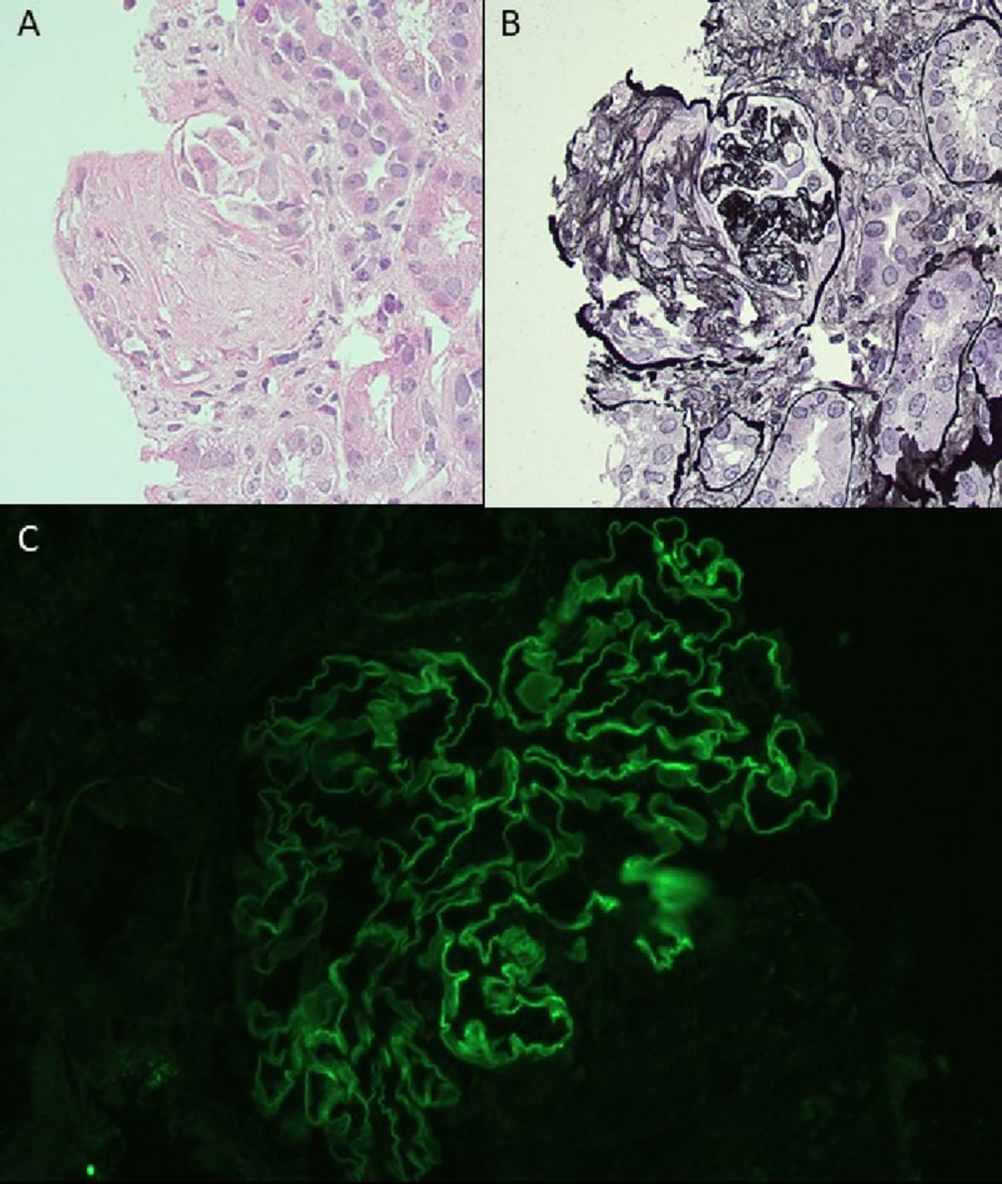
Images from kidney biopsy taken 4-weeks post-diagnosis of anti-GBM disease depicting a fibrocellular crescent. **A **Hematoxylin and eosin stain. **B **Periodic acid–Schiff stain. **C **Immunofluorescence showing linear IgG staining of glomerular basement membrane

The diagnosis was a rapidly progressive glomerulonephritis caused by anti-GBM disease: her serum creatinine had increased tenfold (to 5.98 mg/dL) over the three preceding weeks and anti-GBM titer was 570CU. She had a positive ANA (nucleolar pattern with titer 1:80) and negative dsDNA, ENA, ANCA and cryoglobulins, with normal C3 and C4 levels. The patient received apheresis and immunosuppression with high-dose steroids and intravenous cyclophosphamide. The kidney function continued to decline, hence hemodialysis was commenced (Fig. 1). The anti-GBM titer continued to decrease (Fig. 1). The 4-week kidney biopsy showed healing anti-GBM disease with one-sixth of glomeruli showing crescents and half with partial or global sclerosis (Fig. 2). There were no active cellular crescents.

Maintenance azathioprine was administered for 6 months and the patient was dialysis-dependent for 2 months. At the 1-year review, the serum creatinine improved to 1.69 mg/dL and the anti-GBM titer was undetectable (< 3CU).

Figure 1 highlights two episodes of acute kidney injury occurring after 12 months. These were both in the setting of a urinary tract infection (UTI). The patient’s anti-GBM titer was negative on both occasions and vasculitis screen negative. However, the creatinine never returned to baseline.

Twenty months after the initial event (30-month post-alemtuzumab therapy), the patient, now 24 years, re-presented with uremic symptoms and hematuria. The serum creatinine was 14.14 mg/dL, urea 82.61 mg/dL and anti-GBM titer was 351CU.

**Fig. 3:**
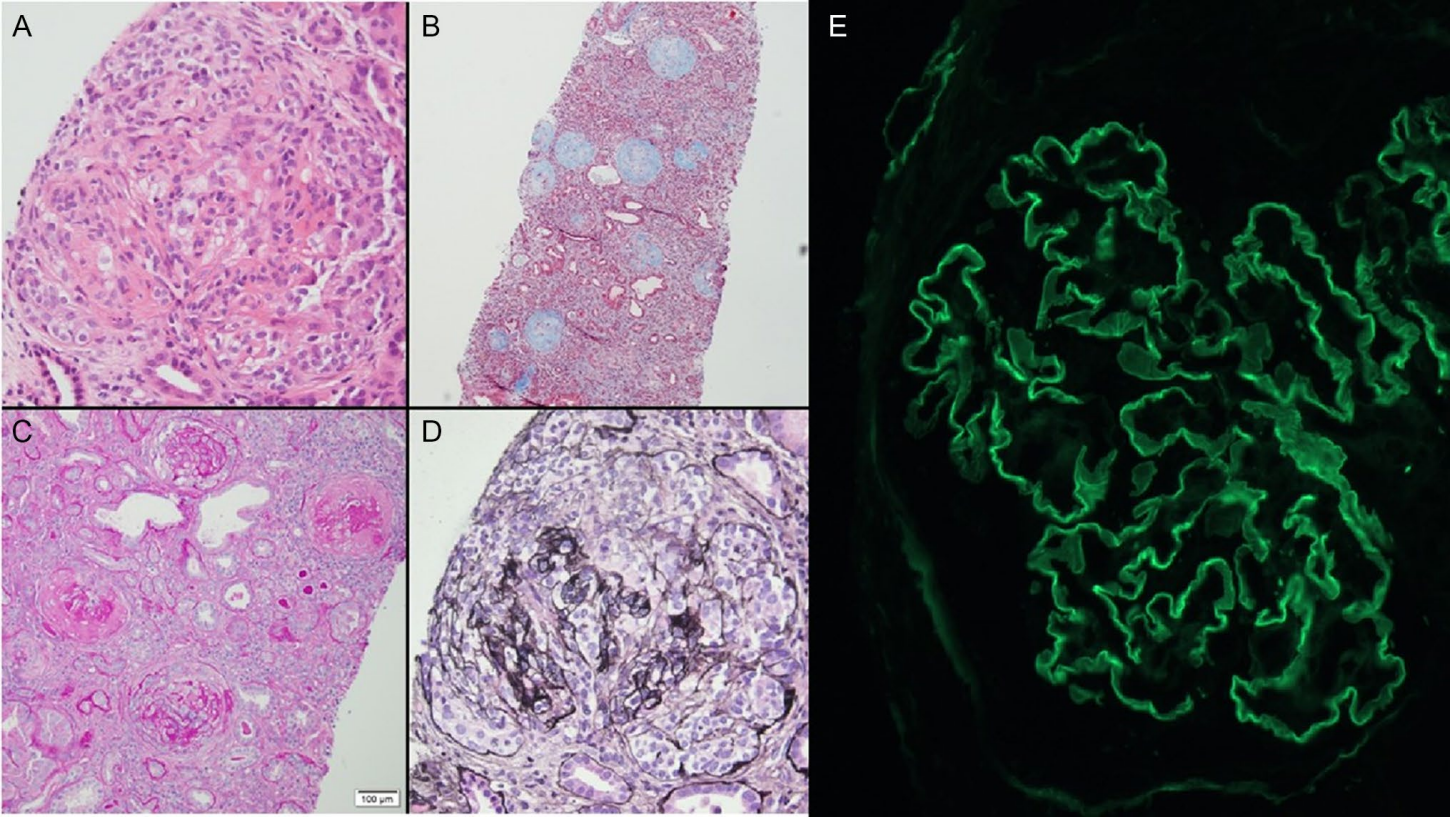
Images from kidney biopsy taken 4-weeks post-relapse of anti-GBM disease depicting crescents. **A **Hematoxylin and eosin stain. **B **Masson's trichrome stain. **C **Periodic acid–Schiff stain. **D **Silver stain. **E **Immunofluorescence showing linear IgG staining of glomerular basement membrane

The relapse of anti-GBM disease was treated with steroids and apheresis, and hemodialysis was re-commenced. Due to the patient preferring not to have cyclophosphamide, intravenous rituximab was given for immunosuppression and subsequently, anti-GBM titers were down-trending (Fig. 1). However, kidney biopsy at 4-weeks post-relapse showed active chronic anti-GBM disease with active cellular crescents in all functioning glomeruli and severe parenchymal scarring (Fig. 3). The patient is now dialysis-dependent. Her kidney transplant work-up revealed positivity for HLA-DRB1*1501.

## Discussion

The etiology of anti-GBM disease is thought to be mainly idiopathic, but environmental triggers such as infection, smoking and hydrocarbons have been implicated [1]. In addition, increased risk is associated with HLA-DR15 and DR4 and decreased risk with HLA-DR1 and DR7. Interestingly, HLA-DR15 is associated with an increased risk of MS, which may be of particular relevance in this case as the patient does carry HLA-DRB1*1501 [1].

A recently identified trigger for anti-GBM disease is alemtuzumab, an anti-CD52 monoclonal antibody approved for the treatment of relapsing–remitting MS [2]. Of all MS treatments, alemtuzumab has the longest lasting effect on the immune system [3].

CD-52 is expressed by T and B cells, monocytes, macrophages, and eosinophils [3]. Alemtuzumab is involved in the selective depletion of T and B cells that express CD-52 mainly in the peripheral blood and less so in the lymphoid tissue [3]. Repopulation occurs within weeks with a relative increase in regulatory T cell production and decreased production of pro-inflammatory cytokines [2]. Alemtuzumab has been shown to induce long-term remission of MS after a short treatment course [4].

Despite this, alemtuzumab is associated with delayed secondary autoimmunity [5]. According to the CARE–MS study, the delayed secondary autoimmunity mostly occurred in the form of autoimmune thyroid disease and then autoimmune thrombocytopenia. Autoimmune kidney disease occurred in 0.2% of patients [1]. Both anti-GBM disease as well as membranous nephropathy have been reported. The frequency of autoimmune kidney disease in patients treated with alemtuzumab worldwide is estimated to be 0.13% [1].

The half-life of alemtuzumab is about 2 weeks, and within 30 days of treatment, serum concentrations are generally undetectable [2]. However, most secondary autoimmune events related to alemtuzumab occur months to years after the last infusion, suggesting the mechanism may be related to the initial depletion and repopulation process occurring early in the treatment timeline [2].

One study suggests that autoimmunity after alemtuzumab develops when homeostatic proliferation of T cells dominates the reconstitution process [6]. Homeostatic proliferation is triggered by lymphopenia [7]. Positive and negative selection of T cells that occurs in the thymus creates a group of T cells with differing affinities for self [7]. Some have relatively high self-affinity, though not strong enough to undergo negative selection. These cells are usually quiescent. However, when T cell depletion occurs, homeostatic proliferation of existing T cells occurs, including those that escaped depletion, so the repertoire of T cells may be skewed towards those with high self-affinity [6, 7]. Unfortunately, there are no markers available to identify the risk of secondary autoimmunity in patients [8]. Another theory is that in the repopulation process, B cells repopulate rapidly without initial T cell regulation, potentially promoting autoimmunity [4].

A literature review revealed 12 previous cases of anti-GBM disease after alemtuzumab treatment (Table 1). There seem to be two peaks of disease occurrence: between three and 10 months after the last dose of alemtuzumab; and around 3 years following alemtuzumab. None of the case reports indicated relapsing disease post-recovery. Therefore, this is the first case report of relapsing anti-GBM disease following remission in the context of alemtuzumab treatment.

**Table 1:** Summary of literature review of previously reported cases of alemtuzumab-related anti-GBM disease

Age/gender	Indication for alemtuzumab	Months after last alemtuzumab	Pulmonary involvement	High anti-gbm titre	Biopsy confirmatory	Treatment	Dialysis requirement	Kidney function recovery	Transplant	Reference
37 Male	MS	3	Yes	Yes	No	Prednisone, oral cyclophosphamide, plasma exchange	Yes	No	No	(5)
35 Female	MS	39	No	No	Yes	Prednisone, cyclophosphamide, plasma exchange	No	Yes	No	(11)
40 Female	MS	9	No	Yes	Yes	Plasma exchange, pulsed cyclophosphamide, and corticosteroids	Yes	No	Yes	(12)
43 Male	Antineutrophil cytoplasmic antibody–associated vasculitis	10	No	Yes	Yes	–	Yes	No	Yes	(12)
35 Female	MS	24	–	–	–	Plasma exchange, mycophenolate and steroids	–	No	Yes	(11)
42 Female	MS	36	No	Yes	Yes	High dose steroids, cyclophosphamide, plasma exchange and rituximab	Yes	No	No	(13)
42 Male	MS	–	No	No	Yes	High dose steroids, mycophenolate, plasma exchange and rituximab	No	Yes	No	(13)
30 Female	MS	5	No	Yes	No	Plasma exchange, rituximab, intravenous methylprednisolone and oral prednisolone	Yes	No	No	(14)
–	MS	–	No	Yes	No	Plasma exchange, cyclophosphamide and intravenous steroid	No	Yes	No	(15)
–	MS	36	No	Yes	–	–	–	Yes	–	(2)
46 female	MS	8	Yes	Yes	Yes	Plasma exchange, prednisone, cyclophosphamide	No	Yes	No	(16)
52 Female	MS	39	No	Yes	Yes	Plasma exchange, oral cyclophosphamide and intravenous steroid	Yes	No	No	(17)

Anti-GBM disease is usually a monophasic condition. Relapse is uncommon and is usually associated with continued exposure to environmental triggers, such as pulmonary irritants [9]. In this case, it could be surmised that after remission was obtained, the two subsequent UTIs may have revealed sequestered epitopes within the GBM causing recurrence of anti-GBM disease in this already susceptible individual [10]. Due to the rare nature of relapsing disease, current guidelines do not even recommend maintenance immunosuppressive therapy.

Given the risk of secondary autoimmunity, and following the death of one patient in a clinical trial, rigorous monitoring post-alemtuzumab has been proposed. One recommendation is to screen patients for serum anti-GBM antibodies regularly. For the first 48-months post-treatment, it is proposed that kidney and hematological functions are screened monthly, and thyroid function is screened every 4 months [3]. Kidney monitoring involves serum creatinine measurements and urine analysis with microscopy [1].

## Conclusion

We present the first case of relapsing anti-GBM disease secondary to alemtuzumab therapy in a patient with multiple sclerosis. Further elucidation of the mechanisms involved in generation of secondary autoimmune reactions with alemtuzumab is required. Nonetheless, it is prudent to monitor these patients with monthly kidney function tests to detect early kidney impairment before rapid progression. Furthermore, close follow-up after remission of anti-GBM disease with regular anti-GBM titer monitoring is crucial to detect relapse before further extensive kidney damage ensues. These measures may decrease morbidity and the likelihood of dialysis-dependence.

## Declarations

### Research involving human and animal participants

This article does not contain any studies with human participants or animals performed by any of the authors.
